# Interaction between tissue-dwelling helminth and the gut microbiota drives mucosal immunoregulation

**DOI:** 10.1038/s41522-023-00410-7

**Published:** 2023-06-24

**Authors:** Yugui Wang, Aijiang Guo, Yang Zou, Wenjie Mu, Shengying Zhang, Zhiqi Shi, Zhongli Liu, Xuepeng Cai, Xing-Quan Zhu, Shuai Wang

**Affiliations:** 1 College of Veterinary Medicine, Shanxi Agricultural University, Taigu, Shanxi Province 030801 China; 2 State Key Laboratory of Animal Disease Control and Prevention, College of Veterinary Medicine, Lanzhou University, Lanzhou Veterinary Research Institute, Chinese Academy of Agricultural Sciences, Lanzhou, Gansu 730000 China; 3 Key Laboratory of Veterinary Parasitology of Gansu Province, Gansu, 730046 China; 4 Jiangsu Co-innovation Center for Prevention and Control of Important Animal Infectious Diseases and Zoonoses, Yangzhou, Jiangsu 225009 China

**Keywords:** Microbiology, Pathogens

## Abstract

Tissue-dwelling helminths affect billions of people around the world. They are potent manipulators of the host immune system, prominently by promoting regulatory T cells (Tregs) and are generally associated with a modified host gut microbiome. However, the role of the gut microbiota in the immunomodulatory processes for these non-intestinal parasites is still unclear. In the present study, we used an extra-intestinal cestode helminth model-larval *Echinococcus multilocularis* to explore the tripartite partnership (host-helminth-bacteria) in the context of regulating colonic Tregs in Balb/c mice. We showed that larval *E. multilocularis* infection in the peritoneal cavity attenuated colitis in Balb/c mice and induced a significant expansion of colonic Foxp3^+^ Treg populations. Fecal microbiota depletion and transplantation experiments showed that the gut microbiota contributed to increasing Tregs after the helminth infection. Shotgun metagenomic and metabolic analyses revealed that the gut microbiome structure after infection was significantly shifted with a remarkable increase of *Lactobacillus reuteri* and that the microbial metabolic capability was reprogrammed to produce more Treg cell regulator-short-chain fatty acids in feces. Furthermore, we also prove that the *L. reuteri* strain elevated in infected mice was sufficient to promote the colonic Treg frequency and its growth was potentially associated with T cell-dependent immunity in larval *E. multilocularis* infection. Collectively, these findings indicate that the extraintestinal helminth drives expansions of host colonic Tregs through the gut microbes. This study suggests that the gut microbiome serves as a critical component of anti-inflammation effects even for a therapy based on an extraintestinal helminth.

## Introduction

Helminths are multicellular parasitic worms that affect one-third of the global population worldwide. They can lead to chronic infections by modulating host type 2 immune responses, thereby avoiding immune-mediated expulsion^1^. Chronic helminth infection has been associated with reduced prevalence of allergy, autoimmunity, and inflammatory bowel disease in human populations or suppression of these diseases in animal models^2^. Therefore, understanding how helminths manipulate host immune immunity is of utmost importance for exploring ways to treat allergic or inflammatory conditions, a growing burden on the healthcare system in developed and even developing countries.

Typically, the modulation of helminth infection on host Th2 immune responses involves the suppression of multiple immune cell types through the release of various of excretory-secretory (ES) products^3^. One of the most prominent pathways for helminth immunomodulation is promoting regulatory T cell (Treg) expansion^4^. Tregs have central roles in preventing immune dysregulation and protecting the host from pathogenic immune responses^5^. Studies have suggested that helminths induce Tregs either directly by secreting factors or indirectly by interacting with bystander cell types such as dendritic cells and macrophages that induce Tregs. In addition, increasing evidence suggests that cross-talk with the local microbiota also has an important role^4,6^. Recently, the causal relationship in which intestinal microbiota contributes to the ability of intestinal helminths to modulate allergic disease or inflammation through promoting Treg expansions has been established^7,8^. Several studies have shown that infection with *Heligmosomoides polygyrus* induces significant alterations in the microbiota^3^ and correlates with the increase in short-chain fatty acid (SCFA) production^8^, which promotes immune regulatory response by stimulating the differentiation and suppressive capacity of Foxp3^+^ Tregs in the intestine^9^.

The mechanisms underlying the induction of Tregs by the gut microbiota have almost exclusively been defined from studies on the intestine-dwelling or submucosa helminth models (i,e., *H. polygyrus*, *Trichuris muris* and *Hymenolepis diminuta*)^8,10–12^. Due to colonization of the intestinal lumen, the reported quantitative and qualitative changes in the gut microbiota composition following these intestinal helminth infections may be associated with direct interplay between the gut microbiota and helminth (or helminth ES products), in which the direct interventions from secretions or collateral mucosal tissue damages might potentially confound the research observations. Tissue-dwelling helminths or helminths with an extra-intestinal phase of their lifecycle, represent a large proportion of the whole helminth population, including many nematodes, cestodes, and trematodes, and can change the host gut microbiota. For example, *Schistosoma mansoni*^13^*, larval* Echinococcus granulosus^14^ and *Trichinella spiralis*^15^ have been reported to be associated with the alterations of the gut microbiota in the host. However, whether these gut alterations participate in mechanisms of Treg differentiation in these tissue-dwelling helminth infections is yet to be ascertained.

In this study, we used an extra-intestinal cestode helminth-larval (metacestode) stage of *Echinococcus multilocularis* to explore whether there is an interaction between tissue-resident parasites and the host gut microbiota to modulate host mucosal immunity through promoting Tregs. *E. multilocularis* is the causative pathogen for alveolar echinococcosis, and its infection in the peritoneal cavity of mice serves as a good experimental model in molecular host-parasite interplay^16^. Our results showed that larval *E. multilocularis* infection could modulate the host mucosal immunity through a cross-talk with the gut microbiota, in which the *Lactobacillus reuteri* isolate is expanded and able to promote Treg expansion. This study established the mutualistic relationship between host gut microbiota and the promotion of Treg cells in a tissue-dwelling helminth.

## Results

### Chronic *Echinococcus multilocularis* infection reduces colitis in Balb/c mice

**Fig. 1:**
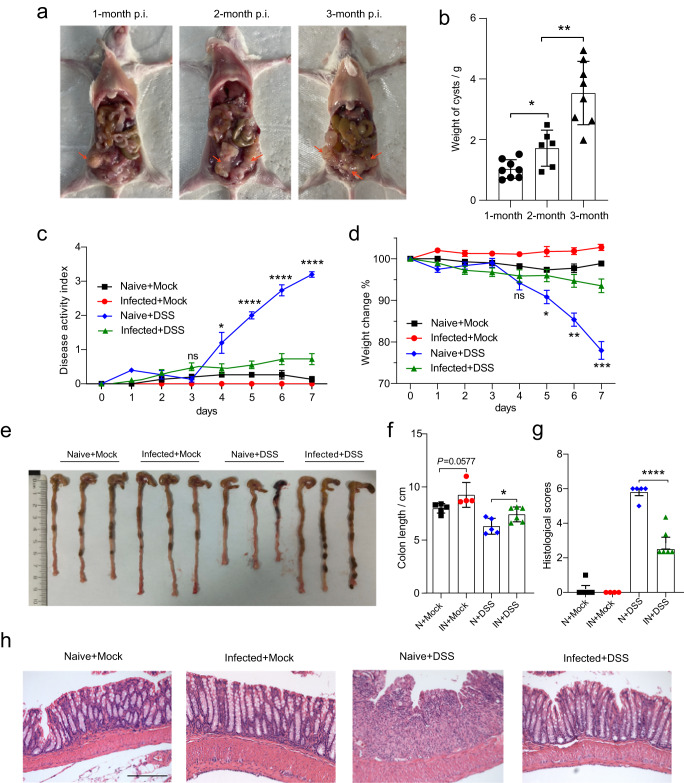
*E. multilocularis* infection reduces DSS-induced colitis in Balb/c mice. The experiments were conducted on mice on day 90 post-infection (p.i.) of *Emu* in the peritoneal cavity (panels (**a**, **b**)). The Balb/c mice were treated with either 5% DSS or Mock (autoclaved water) for 7 days. Larval *E. multilocularis* infection (on day 90 post-infection) in the peritoneal cavity attenuated the colitis in Balb/c mice (N Naïve, IN Infected), in the context of disease activity index (DAI) (**c**), weight loss across the experiment (**d**), colon length (**e**, **f**), inflammation scores estimated from distal colon tissue with H&E staining (**g**), and representative H&E staining sections of distal colon tissues (×100; The scale bar represents 200 µm in length) (**h**). Experiments were repeated 2 times with similar results. The data are shown from a representative experiment (mean ± SD shown). *P < 0.05, **P < 0.01, ***P < 0.001, ****P < 0.0001 using the two-sided Student *t*-test (*n* = 6–8 mice per stage for panel (**b**) and *n* = 4–6 per group for panels (**c**–**h**)).

Consistent with the findings of previous studies suggesting that helminth infections are associated with remission of intestinal inflammation^1,17^, we observed that *E. multilocularis* larvae (*Emu*) infection reduced intestinal mucosal damage and inflammatory responses in DSS-induced colitis in the Balb/c mouse model (Fig. 1). Inflammation for the Balb/c mice at 90 days post-*Emu* infection (Fig. 1a, b) was triggered by dextran sodium sulfate (DSS) administration in drinking water. As expected, DSS treatment resulted in a significantly higher Disease Activity Index (DAI) score (Fig. 1c), reduced weight (Fig. 1d), shortened colon length (Fig. 1e, f), and mucosal damage and mucosal erosion (Fig. 1g, h) in the naive group. In contrast, the mice infected with *Emu* suffered a less severe inflammation with a reduction in weight loss, disease activity, colon shortening and pathologic damages (Fig. 1c–h). These results indicate that the infection of this extra-intestinal worm-larval *E. multilocularis* is associated with colonic immunoregulation and reduction of colitis in this model.

### Chronic *Emu* infection induces expansion of colonic regulatory T cells

**Fig. 2:**
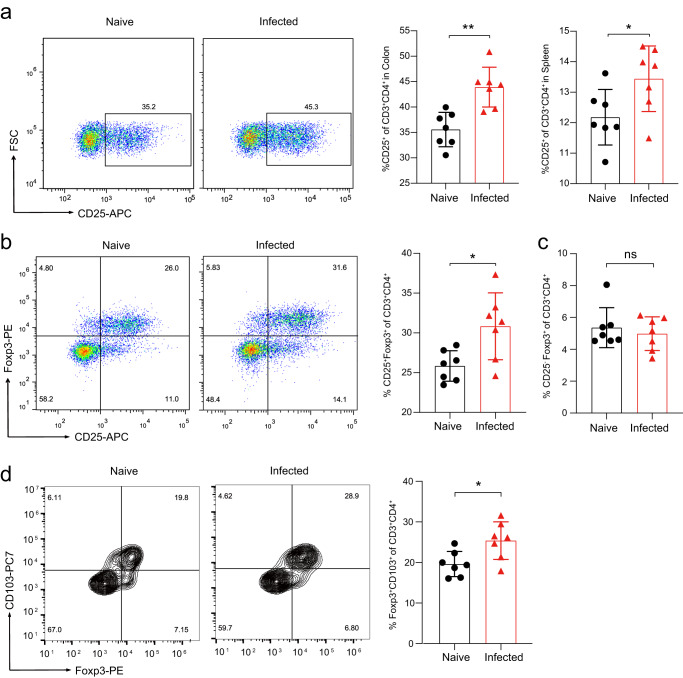
*E. multilocularis* infection promotes colonic Treg expansion. On day 90 post-infection of *Emu*, the mice were euthanized and subject to flow cytometry analysis of colonic (lamina propria) and splenic lymphocytes. **a** Expression of CD25 by immune cells isolated from the colon of naive and *Emu*-infected mice (left), and proportions of CD25^+^ T cells in CD3^+^CD4^+^ cells in colon or spleen (right). **b** Expression of CD25 and Foxp3 by immune cells isolated from the lamina propria of the colon in naive and *Emu*-infected mice (left), and the proportion of colonic CD25^+^Foxp3^+^ T cells in the CD3^+^CD4^+^ cell population (right). **c** The proportion of colonic CD25^-^Foxp3^+^ T cells in the CD3^+^CD4^+^ cell population. **d** Expression of CD103 and Foxp3 by immune cells isolated from the lamina propria of the colon in naive and *Emu*-infected mice (left), and the proportion of colonic CD103^+^Foxp3^+^ T cells in the CD3^+^CD4^+^ cell population in LP (right). Flow cytometry data are representative of *n* = 7 mice per group. Experiments were repeated 2 times with similar results. The data shown are from a representative experiment (mean ± SD shown). ns, not significant. *P < 0.05, **P < 0.01, using the two-sided Student *t*-test.

Tregs have central roles in helminth-mediated gut immune regulation^18,19^. We found that CD4^+^ T cells from *Emu*-infected mice at day 90 post-infection were oriented towards a Treg pathway (CD4^+^CD25^+^) at either spleen or colon site (Fig. 2a). Forkhead box P3 positive (Foxp3^+^) Treg cells are the pivotal Treg populations that are critical for immune homeostasis and inflammation control. In keeping with the data in the DSS-induced model, Tregs at lamina propria (LP) of the colon site showed a significantly higher proportion of T cells with the expression of Foxp3^+^ (CD4^+^CD25^+^Foxp3^+^; Fig. 2b and Supplementary Fig. 1), indicating that the developments of Tregs following *Emu* infection were promoted at the colon site. Specifically, the frequencies of CD4^+^CD25^high^Foxp3^+^ T cells but not CD4^+^CD25^low^Foxp3^+^ were significantly elevated in these mice (Fig. 2c). CD103 is a well-established marker for murine effector/memory-like Treg cells which is expressed following their activation in infectious diseases^20^. We observed a marked increase in the proportion of colonic CD103^+^Foxp3^+^ Treg cells within the CD4^+^ T cells in the *Emu*-infected mice (Fig. 2d). Collectively, these data suggest that *Emu* infection potentially raises the activated colonic Foxp3^+^ Treg cell populations.

### The gut microbiota promotes Treg cells differentiation in *Emu* infection

**Fig. 3:**
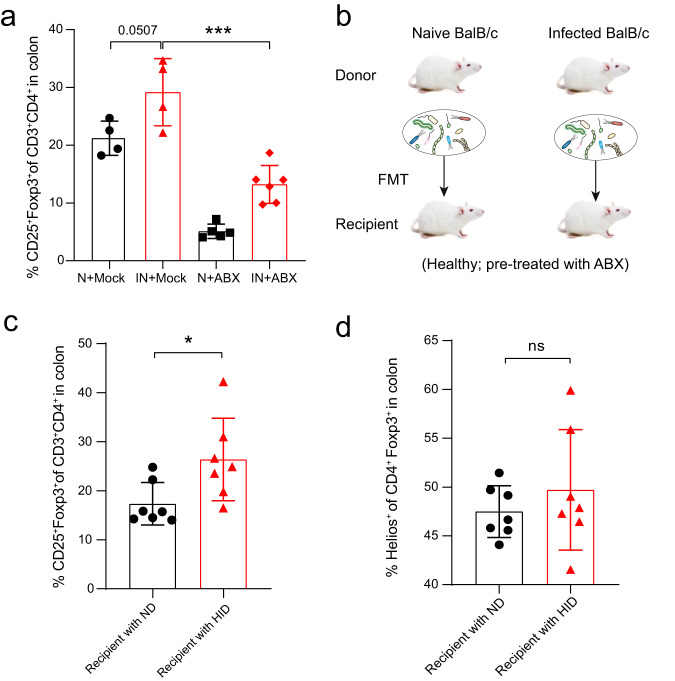
The gut microbiota is associated with Treg cell differentiation in *Emu* infection. **a** After depletion of the gut microbiota with antibiotic cocktail treatment (ABX), the proportion of colonic CD25^+^Foxp3^+^ Treg cells in the CD3^+^CD4^+^ cell population in *Emu*-infected (IN) mice was significantly decreased in comparison with that in the infected mice treated with Mock. Naive mice (N) with or without ABX treatment were included as controls. **b** Schematic showing the experimental design for fecal transplantation transfer (FMT). Recipient mice were pretreated with ABX cocktails for 2 weeks, followed by an FMT regime of 2 weeks from naive donors (ND) or helminth-infected donors (HID). **c** The proportion of colonic CD25^+^Foxp3^+^ Treg cells in the CD3^+^CD4^+^ cell population by flow cytometry analysis for the recipient mice. **d** The proportion of colonic Helios^+^ Treg cells in Foxp3^+^ Treg cells by flow cytometry analysis for the recipient mice. The flow cytometry analysis was performed at 4 weeks post-FMT engraftment. Experiments were repeated at least 2 times with similar results. The data shown are from a representative experiment (mean ± SD shown). ns, not significant; *P < 0.05, ***P < 0.001 using the two-sided Student *t*-test (*n* = 4–6 mice for panel **a**; *n* = 7 for panels (**b**–**d**)).

As the gut microbiota is a potent inducer of intestinal Foxp3^+^ Treg cells in the colonic mucosa^21^, we next tested whether the gut microbiota contributes to the expansion of colonic Foxp3^+^ Treg during *Emu* infection. First, we depleted the gut microbiota of Naive or *Emu*-infected mice with an antibiotic cocktail (ABX) regime and analyzed the colonic Treg populations in these mice. The antibiotic treatment significantly depleted the gut microbes in these mice (Supplementary Fig. 2). The colonic Foxp3^+^ Treg (or CD4^+^CD25^+^) ratio within CD3^+^CD4^+^ T cell population in the infected mice with ABX treatment was significantly reduced in comparison with the infected mice treated with vehicle control, albeit higher than that in naive mice with ABX treatment (Fig. 3a). These data suggest that the proliferation of Foxp3^+^ Treg cells induced by infection with *Emu* is tightly linked with the gut microbiota.

We therefore further investigated the role of gut microbes through fecal microbiota transplantation (FMT). We transferred the fecal microbiota of either naïve or *Emu*-infected mice at day 90 post-infection into groups of healthy mice that had been pretreated with an antibiotics cocktail for 2 weeks (Fig. 3b). As the tissue-resident parasite lives in the peritoneal cavity of the infected mice, no parasite was transferred. At 4 weeks after FMT engraftment, the colonic Foxp3^+^ Treg frequency in the recipient mice that were transplanted with the gut microbiota from helminth-infected donors (HID) was significantly enhanced in comparison with that in the mice treated with the gut microbiota collected from the naive donors (ND) (Fig. 3c). These data suggest that the gut microbiota contributes to the expansion of colonic Foxp3^+^ Tregs in the mice with *Emu* infection. Previous studies have shown that gut microbiota induces the expansions of Tregs already present in the colonic LP, regardless of its origin^9^. In accordance with this finding, we observed a comparable frequency of Tregs that co-express Helios (Helios^+^, which indicates a thymic origin) between recipient mice transferred with the gut microbiota from ND and HID (Fig. 3d). Interestingly, the gut microbiota from HID failed to induce a higher proportion of CD25^+^Foxp3^+^ Tregs (Supplementary Fig. 3) in the spleen when compared with that in the mice transplanted with microbiota from ND. Taken together, these results suggest that the gut microbiota promotes intestinal mucosal Treg expansions during larval *E. multilocularis* infection.

### The gut microbiome is skewed with increased *L. reuteri* after *Emu* infection

**Fig. 4:**
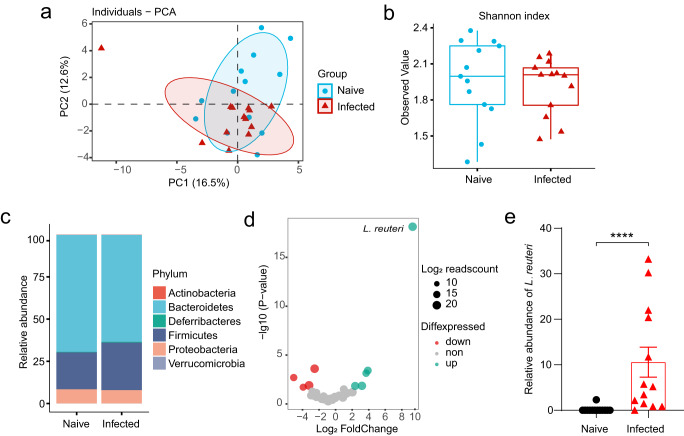
The gut microbiome is altered after *Emu* infection. The shotgun metagenomic analysis of fecal samples was collected from the naive (*n* = 13) or *Emu*-infected mice (*n* = 13) on day 90 post-infection. **a** Principle component analysis (PCA) for the gut microbiome of the samples based on the relative abundance of each taxon at the species level (R2 = 0.14, *P* = 0.003). **b** Shannon index of alpha diversity (*P* > 0.05). **c** The relative abundances of taxa at the Phylum level. **d** Differential abundance analysis of the gut microbiota components at the species level. Log10-transformed *P*-values are shown on Y-axis. The colors of the points indicate whether a taxon in the *Emu*-infected group was over-represented (up), under-represented (down) and unchanged (non) in comparison with that in the naive group. **e** The relative abundance of *L. reuteri* (mean ± SD shown). The significance of PCA analysis for panel a was determined by Permutational multivariate analysis of variance (PERANOVA). ****P < 0.0001 using the two-sided *Mann-Whitney*-test for panels (**b**, **e**). Data in boxes of panel (**b**) represent the 25th percentile, median, and 75th percentile and whiskers stretch to 1.5 times the interquartile range from the corresponding hinge.

The above results strongly suggest that the extraintestinal helminth infection induced alterations in the gut microbiota. Next, we used shotgun metagenomics to compare the species-level microbiome compositions of the fecal samples collected from the naive mice and *Emu*-infected mice at day 90 post-infection. Our data revealed that *Emu* infection induced significant compositional changes in the gut microbiota (Fig. 4a–d). As indicated by the Principal component analysis (PCA) (Fig. 4a, P < 0.01, PERANOVA test), the microbiota community structure in infected mice was significantly skewed in comparison with that of naive mice, whereas no significant changes in alpha diversity were observed (Fig. 4b). At the phylum level, the *Emu* infection induced an expansion of Firmicutes and a reduction of Bacteroidetes (Fig. 4c). At the species level, the differential analysis revealed that *L. reuteri* was the most differentially abundant bacterium to discriminate naive against infection (Fig. 4d). The relative abundances of *L. reuteri* were much higher in mice infected with *Emu* than in naive, with an average fold-change of more than 100 (Fig. 4e). These data suggest that *Emu* infection resulted in a significant alteration of the gut microbiota and a striking expansion of *L. reuteri*.

### Metabolic reprogramming of the gut microbiota increases short-chain fatty acids

**Fig. 5:**
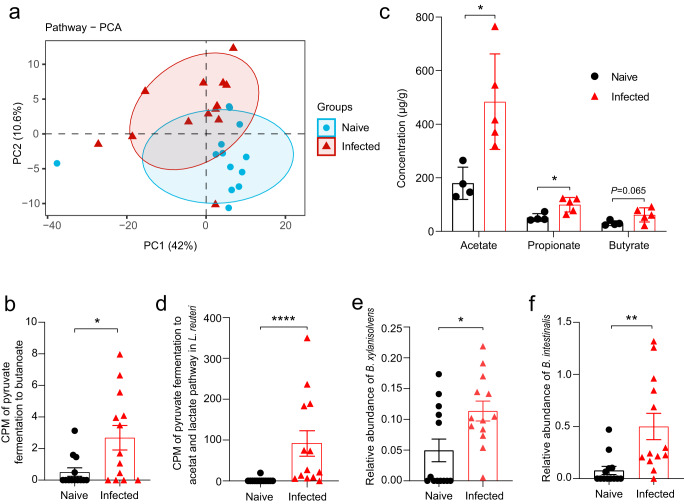
Reprogramming of metabolomic capability after *Emu* infection. The abundance of each pathway in the shotgun metagenomic analysis was evaluated by HUMAnN2. **a** Principle component analysis (PCA) for the gut microbiome based on the relative abundance of each pathway at the species level (R2 = 0.22, *P* = 0.01). **b** Counts per million (CPM) of the pathway of ‘pyruvate fermentation to butanoate’ of the microbiome in the MetaPhlan analysis. **c** The concentrations of short-chain fatty acids of fecal samples in the metabolomic analysis (mean ± SD shown). **d** CPM of the pathway of ‘pyruvate fermentation to acetate and lactate’ in an *L. reuteri* isolate in the MetaPhlan analysis. **e** Relative abundances of *Bacteroides xylanisolvens* and (**f**) *Bacteroides intestinalis*. The significance of PCA analysis for panel a was determined by Permutational multivariate analysis of variance (PERANOVA). ns, not significant. *P < 0.05, **P < 0.01, ****P < 0.0001 using the two-sided *Mann-Whitney*-test for panels (**b**), and (**d**–**f**) (*n* = 13 for naïve mice and *n* = 13 for infected mice), and using the two-sided Student-*t* test for panel (**c**) (*n* = 4–5 per group).

We wondered if the changes in microbial community structure would translate into altered microbial metabolism. PCA of metabolic pathway abundance data for the gut microbiome showed a significant separation (*P* < 0.05) between animals uninfected and infected with *Emu* (Fig. 5a). Differential abundance analysis identified 38 global pathways and 93 pathways at the species level that were significantly altered after infection (Supplementary Data 1) (*FDR* < 0.05), including several involved in carbohydrate metabolism and short-chain fatty acids (SCFAs) synthesis (*e.g*., ‘pyruvate fermentation to butanoate’ Fig. 5b). Of note, microbiota-derived short-chain fatty acids (SCFAs) are well-known anti-inflammatory mediators and Treg inducers for host intestinal immunity^9^. Using targeted metabolomics, we measured the levels of seven SCFAs in the stool samples of the mice. Consistent with observations in the in silico metagenomic analysis, we found that levels of fecal SCFAs (acetate, propionate, and butyrate) were significantly elevated in mice infected with *Emu* (Fig. 5c), indicating an enhanced capability of SCFAs formation of the gut microbiota in the *Emu*-infected mice.

Specifically, the metabolic profiling at the species level based on shotgun metagenomic data revealed that the pathway of ‘pyruvate fermentation to acetate and lactate’ in an *L. reuteri* isolate was significantly increased in abundance (Fig. 5d). Although *L. reuteri* might generally not be capable of direct formation for propionate and butyrate, it facilitates the production of these SCFAs by providing acetate and lactate as substrates for colonic SCFA producers, e.g., *Bacteroides spp*^22^. Consistent with this notion, we observed an increase of *Bacteroides* species (i.e., *Bacteroides intestinalis* and *Bacteroides xylanisolvens*) after *Emu* infection (Fig. 5e, f). We isolated the elevated *L. reuteri* strain (confirmed by 16 S rRNA sequencing) from *Emu*-infected mice and then gavaged the *L. reuteri* isolate to healthy Balb/c mice. SCFAs were significantly increased in the feces after the administration (Supplementary Fig. 4). Collectively, the results suggest that an *L. reuteri*-related metabolic shift and increased SCFA levels are hallmarks of the gut microbiome after *Emu* infection.

### *L. reuteri* isolate enhances Treg frequencies and is promoted by *Emu* infection

**Fig. 6:**
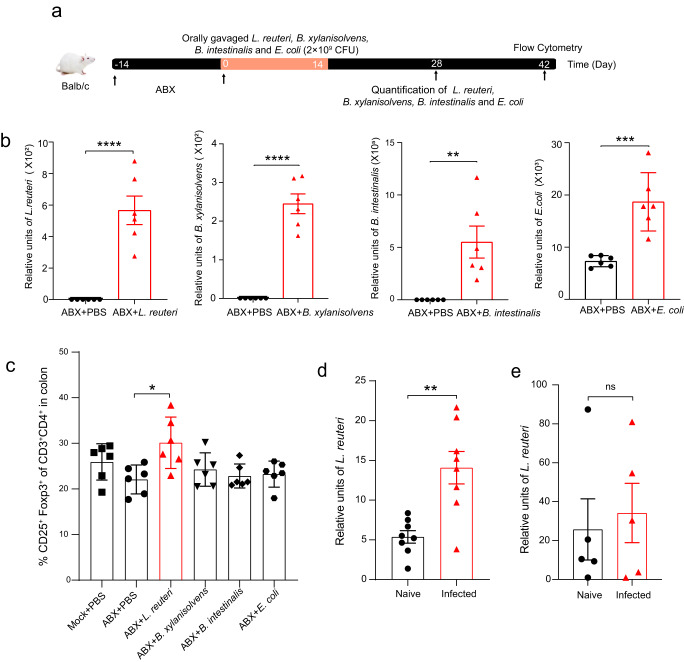
The *L. reuteri* isolate enhances Treg frequency and is promoted by *Emu* infection. **a** The schematic showing the experimental design for *L. reuteri* and other control microbe administration experiments. The mice were orally gavaged with each isolate daily for 2 weeks (on days 0–14) after antibiotics (ABX) treatment. **b** The level of each bacterium was measured by qPCR on day 28. **c** The frequency of colonic Foxp3^+^ Treg cells was quantitatively measured by Flow cytometry on day 42. The *L. reuteri* isolate (2 × 10^9^ CFU per mouse) was administrated to the mice on day 60 post-*Emu* infection for panels d-e and the levels of the bacterium in feces were detected. **d** The level of *L. reuteri* in naive or *Emu*-infected Balb/c mice. **e** The level of *L. reuteri* in naive or *Emu*-infected Nude Balb/c mice. Data for each sample are presented as the mean expression level in arbitrary units. Experiments were repeated at least 2 times with similar results. The data shown are from a representative experiment (mean ± SD shown). ns, not significant. *P < 0.05, **P < 0.01, ***P < 0.001, ****P < 0.0001 using the two-sided Student *t*-test for panels (*n* = 6 mice per group for panels **b**, **c**; *n* = 8 for panel **d**; *n* = 5 for panel **e**).

Together with evidence from microbiota depletion and FMT experiments (Figs. 3 and 4), these data suggest that the microbial alterations marked by *L. reuteri* expansions contribute to the elevation of Tregs. In accordance with the notion, we found that the Tregs ratio was not altered 6 weeks after infection (Supplementary Fig. 5), where the gut microbial structures (Supplementary Fig. 6a) and the relative abundance of *L. reuteri* were slightly changed (Supplementary Fig. 6b), which indicates that alterations in microbiota and *L. reuteri* occurred earlier than the Treg expansions. Although certain *L. reuteri* strains have been shown to have the capability to promote Tregs^23,24^, a high variation for this capability usually exists among bacteria isolates. To test whether the isolated *L. reuteri* strain could promote Tregs, this bacterium and other controls (*Escherichia coli*, *B. intestinalis*, and *B. xylanisolvens*) were administrated to uninfected mice by gavage for 2 weeks (Fig. 6a). qPCR analysis of fecal samples showed successful colonization of these bacteria isolates in the gut of these mice after administration (Fig. 6b). The frequency of Foxp3^+^ Tregs was significantly elevated in the mice treated with the *L. reuteri* isolate. However, this Treg expansion was not observed in the mice administrated with any other bacteria (i.e., *Escherichia coli*, *B. intestinalis*, and *B. xylanisolvens*) (Fig. 6c). Hence, this data suggests that exposure of the *L. reuteri* isolate alone is sufficient to promote Treg frequencies in the host.

The above data indicate that *L. reuteri* is associated with the host gut immunoregulation after an extra-intestinal helminth infection. Interestingly, we observed that the *L. reuteri* expansion might be promoted by host immunity in return. Naive and *Emu*-infected Balb/c mice received *L. reuteri* isolate by oral gavage (on day 60). We detected the level of *L. reuteri* in the mice post gavage and found that the *L. reuteri* isolate level was much higher in the mice that were *Emu*-infected (Fig. 6d). In contrast, we did not observe a significantly increased level of the *L. reuteri* isolate in the *Emu*-infected Balb/c Nude mice (which are T cell-deficient and were treated the same way) when compared with their naive counterpart (Fig. 6e). These preliminary results raise a possibility that T cells might be linked with the *L. reuteri* overgrowth in the gut of *Emu*-infected mice.

## Discussion

Tregs play a central role in the immunomodulatory effect of helminths^2^. The mechanisms underlying the inductions of Tregs by helminth are only partially understood. Increasing evidence shows the essential contribution of commensal microbiota to the induction and accumulation of the colonic Treg cell population^25^. Currently, the causal link between the gut microbial commensals and the regulatory T-cells in helminth infection has been mainly established in the intestinal helminth model species *H. polygyrus*^7,8,26^. Tissue-dwelling helminths or helminths with an extra-intestinal phase of their life cycles, such as filarial parasites, flukes and tapeworms, represent a large proportion of the helminth population and affect billions of people worldwide^27^. Therefore, it is of utmost importance and interest to dissect the roles of gut microbes in the immunoregulatory effects conferred by these parasites. In this study, we reported that the gut microbiota influences Treg populations, even in an extra-intestinal helminth. Therefore, our results suggest that the involvement of gut microbiota is likely a universal mechanism in helminth-induced regulatory immune responses.

One of the most striking findings in this study is the establishment of the causal link between the expansion of colonic Foxp3^+^ Tregs and the gut microbiota for a tissue-dwelling helminth. In spite of substantially different modes of interaction with the gut mucosa (e.g., luminal vs. tissue-dwellers) for these helminths^3,28^, one aspect that is often overlooked is that helminth-associated changes in gut microbiota composition occur^29^. It is very likely that the three-way interactions universally occur between helminth parasites, the resident microbes, and the host gut. In this study, we confirmed this notion in the context of mucosal immunity in a larval cestode infection in Balb/c mice. Consequently, more consideration needs to be taken on the effect of the gut microbiota when evaluating the influence and therapeutic potential of an extra-intestinal helminth. In addition, the findings observed from this tissue-dwelling helminth support that the shift in commensal microbiota is more likely (at least partially) the response to helminth infection rather than directly driven by helminth secretions or collateral tissue damage. Our results (Fig. 2a) also suggest that multiple cooperating mechanisms between helminth and the gut microbiota might drive the mucosal immunoregulation during helminth infection, as the Tregs were still expanded even after antibiotics treatment during *Emu* infection. This is anticipated and consistent with the findings in extensive previous studies that helminth excretory-secretory (ES) products or induced-cytokines (e.g., TGF-β) are able to directly induce naive CD4^+^ T-cells to become Tregs^4^.

In this study, we observed that the microbial shift is marked by an expansion of an *L. reuteri* isolate. Our results indicate that the *L. reuteri* isolate is able to promote SCFA levels and Treg expansions. Although SCFAs are well-known strong inducers of Tregs, these findings are not sufficient to establish a causal link between *L. reuteri* and Treg expansion during *Emu* infection. Further research is needed to fully clarify this issue. In fact, the expansion of lactobacilli populations upon helminth colonization is one of the most frequently reported observations in different interaction systems between host and parasite species, such as *H. polygyrus* in mouse^7,17^, and *T. spiralis* in mice^15^, *Ascaris suum* in pig^30^, and *Toxocara cati* in cat^31^. Of note, certain *Lactobacillus* species or strains have been reported to exert beneficial therapeutic effects on many autoimmune-related diseases (e.g., allergic airway response, asthma, and allergy contact dermatitis) by augmenting host regulatory T cells^23,32,33^. In addition, the expansion of a *Lactobacillus* species (*L. taiwanensis*) was proved to promote the establishment of *H. polygyrus* infection via the enhancement of Treg-mediated responses in C57BL/6 mice^7^. These data suggest that the cross-talk with lactobacilli commensals is potentially a universal strategy employed by helminths to promote their persistence for long-term survival under host immune system attack, which in turn brings beneficial effects to their hosts under inflammatory or pathogenic conditions. In agreement with this view, an alteration of microbiota-derived immunoregulatory metabolites in helminth infection is also frequently observed in many hosts parasitized by various helminths^3^. Among them, SCFAs are prominent in influencing multiple facets of immunity, including stimulating the differentiation and suppressive capacity of Foxp3^+^ Tregs in the intestine^9^. Helminth infection has been repeatedly reported to affect host carbohydrate metabolism for SCFAs, such as infection of *H. polygyrus* in mice^8^, *Haemouchus contortus* in goats^34^, and *T. suis* in pigs^35^. The findings in this study provide further support that SCFAs potentially act as common microbiota-derived signaling involving extraintestinal helminth-induced regulatory T-cell expansions. In this regard, these metabolites of gut microbiota appear to serve as messengers to facilitate cross-talk between the host and gut microbiota during helminth infections, which may be employed as a strategy to invade host immune systems and hence are profoundly implicated in host health and disease. However, the mechanism through which the helminth infection drives the changes in the microbiome, lactobacilli populations, and SCFAs is still not fully understood. Our results in the nude mice highlight the possibility that this expansion is likely T-cell-dependent. In accordance with this notion, the gut microbiome alterations during *H. polygyrus* and *T. muris* infections were observed to be Th2-associated^10,36^. Thus, the helminth infections might provide an immunity-dependent intestinal niche within the ecosystem of gut microorganisms in favor of the growth of these bacteria. In Fig. 6, L. *reuteri* seems to be much easier to colonize in the gut of Balb/c-Nude mice than in the normal Balb/c mice, which suggests that the absence of T cell-based immunity favors *L. reuteri* expansion. It is noteworthy that both nude mice and helminth-infected mice have impaired T-cell-based immunity and both have higher levels of *L. reuteri*. Thus, this observation together with the findings on *Emu*-infected mice is consistently in agreement with the notion that suppression or loss of T cell-based immunity might be linked with *L. reuteri* expansion. However, the initial gut microbiota structure might have effects on these preliminary observations and the exact mechanisms behind this observation remain elusive.

Overall, the results from this study demonstrated that larval *E. multilocularis* infection induces significant alterations in the host gut microbiome, which is marked by an increase in *L. reuteri* and SCFAs, and that these *Emu*-induced microbial changes contribute to the expansion of colonic Tregs induced by a tissue-dwelling helminth. These findings provide perspectives into the mutualistic relationships between a commensal microbe and a tissue-dwelling helminth parasite in terms of gut immunoregulation. A better understanding of the role of the gut microbiota in helminth-induced immune modulation would facilitate the development of novel therapeutics for inflammatory and autoimmune diseases that have important public health implications.

## Methods

### Mice and infections

Specific-pathogen-free (SPF) Balb/c mice (female, 7–9 weeks old) were purchased from the Laboratory Animals Center of Lanzhou Veterinary Research Institute, Chinese Academy of Agricultural Sciences. SPF Balb/c-Nude mice (Balb/c, C000103, female, 7–9 weeks old) were obtained from Changzhou Cavens Lab Animal Co., Ltd (Jiangsu Province, China). All the animals were housed in an environment with a temperature of 22 ± 1 °C, and a light/dark cycle of 12/12 h. All animal studies (including the mice euthanasia procedure) were done in compliance with the regulations and guidelines of the Institutional Animal Care and Use Committee of Lanzhou Veterinary Research Institute (Reference NO. LVRIAEC-2019-020).

The *E. multilocularis* strain, isolated from Qinghai in China, was used in this study^37^. The *E. multilocularis* protoscolex (metacestode stage) was collected from hydatid cysts isolated from Mongolian gerbils (*Meriones unguiculatus*) that were intraperitoneally inoculated with larval *E. multilocularis* for five months according to the method previously reported^38^. Briefly, the metacestode tissue was cut into thin slices and strained through a tea strainer, followed by three washes with PBS supplemented with 1% penicillin/streptomycin. The larval *E. multilocularis* infection group for each experiment was intraperitoneally injected with *E. multilocularis* protoscolex (*n* = 2000) in 200 μL phosphate-buffered saline (PBS), and the mice in the control group were intraperitoneally injected with the same volume of PBS. The cysts generally live in the peritoneal cavity of the infected mice.

### Dextran sodium sulfate-induced colitis and histology

To induce colitis, the Balb/c mice were given 5% (w/v) dextran sodium sulfate (DSS, MP Biomedicals) (MW = 36,000–50,000) in drinking water for 7 days. The DSS-containing drinking water was refreshed every three days and replaced with autoclaved water normally used in the animal facility on day 7. Mice in the control group were given autoclaved water only. Disease activity index (DAI) was calculated daily, and included loss of body weight data (0, 0–1%; 1, 1–5%; 2, 5–10%; 3, 10–15%; 4, 15–20%), stool performance (0, normal; 1, soft and shaped; 2, loose; 3, between; 4, diarrhea), and the presence of occult blood (0, no blood; 1, +; 2, ++; 3, +++; 4, ++++). The presence of occult blood was measured using the Fecal Occult Blood Test Kit (Leagene, China) according to the manufacturer’s protocol. All the mice were euthanized on day 8. At sacrifice, colons were removed and measured in length. Approximately 1 cm of the distal colon was embedded into paraffin, after fixation in 10% buffered formalin at room temperature for 24 h. Colon sections were stained with hematoxylin and eosin (H&E) to examine histological alterations. Histological scores were evaluated using the criteria previously described^39^. Histology scoring was performed in a blinded fashion with a protocol as follows: cell infiltration: occasional inflammatory cells in the lamina propria (LP) (score 0); increased infiltrate in the LP predominantly at the base of crypts (score 1); confluence of inflammatory infiltrate extending into the mucosa (score 2); transmural extension of infiltrate (score 3). Tissue damage: no mucosal damage (score 0); partial (up to 50%) loss of crypts in large areas (score 1); partial to total 50–100% loss of crypts in large areas, epithelium intact (score 2); total loss of crypts in large areas and epithelium lost (score 3).

### Antibiotic treatments and fecal microbiota transplantation (FMT)

A cocktail of antibiotics (ABX) (ampicillin, vancomycin, gentamicin, neomycin, and metronidazole) (Sigma-Aldrich, USA) was used to effectively eliminate intestinal bacteria^40^. The efficiency of antibiotics treatment was assessed by evaluating the total bacterial load in feces. Briefly, the genomic DNA of fecal samples was extracted using a QIAamp PowerFecal Pro DNA Kit (QIAGEN, Germany). DNA concentration was quantified using a Qubit 4 Fluorometer (Thermo Scientific) and a total load of bacteria was detected by 16 S denaturing gradient gel electrophoresis and determined by qPCR targeting the 16 S rRNA gene (primers F: 5′ -CGGYCCAGACTCCTACGGG-3′; R: 5′-TTACCGCGGCTGCTGGCAC-3)^41^, using the method in a previous study^42^. The qPCR procedure was performed using the SYBR Green-based method of GoTaq® qPCR Master Mix Kit (Promega, USA) according to the manufacturer’s recommendation (Tm = 60 °C). Reactions were set up in triplicate and run alongside a serially-diluted pool of all samples to be analyzed to create a standard curve of Ct value vs. 16 S rRNA gene level in arbitrary units. Positive and negative controls were run on each plate. Data for each sample are presented as the mean level in arbitrary units and normalized by sample weight. To test whether the feces from ABX-treated mice lost the ability to produce SCFAs, which was reflected by inability to change the color of pH-sensitive Bromocresol purple agar (BCP) plates, feces collected from ABX-treated mice were homogenized, diluted, and cultured on BCP in an anaerobic chamber at 37 °C for 24 h. BCP is used to indicate acid production from whatever fermentable substrate is present in the medium. Yellow haloes are produced around acid-producing colonies.

For the gut microbiota depletion experiment in the mice pre-infected with *Emu*, the mice were treated by a protocol based on the 3 R principle. The antibiotic regimen (*ad libitum* in drinking water with 2.5% sucrose; ampicillin 1 g/L, vancomycin 0.5 g/L, gentamicin 1 g/L, neomycin 1 g/L, and metronidazole 1 g/L) was given on day 3 post-*Emu*-infection and with a regimen for 7 consecutive days alternated with 7 days without treatment, which allowed the animals to recover. The mice were euthanized after three months post-*Emu*-infection. For the FMT experiments, recipient mice were pre-treated with the above antibiotic cocktails (by oral gavage; ampicillin 5 g/L, vancomycin 2.5 g/L, gentamicin 5 g/L, neomycin 5 g/L, and metronidazole 5 g/L; dissolved in drinking water) for 2 weeks with a volume of 10 μL/g body weight once per day. The fecal pellets collected from donor mice (naive or infected with *Emu* for 90 days) were diluted in PBS (100 mg/mL), filtered using 70 μm cell strainers (NEST), and administered to recipient mice (healthy) immediately after preparation. The recipient mice received 3 microbiota transfers per week over 2 weeks. The mice were euthanized and subject to colonic Treg detection by flow cytometry at 4 weeks post-FMT engraftment.

### Lymphocyte isolation and flow cytometry

Lymphocytes were isolated from lamina propria of the colon according to previous protocols^43,44^. Briefly, colon tissues were removed from mice, cut open longitudinally, and washed in PBS. The gut content, fat tissues and colonic patches (CPs) were removed. The retained colon tissues were then cut into small pieces (~2 cm) and washed on a shaker in PBS containing 1 mM DTT for 10 min at 37 °C. After that, the colon tissues were incubated twice with shaking in PBS containing 30 mM EDTA at 37 °C for 10 min; the fluid was refreshed for each time. Then, the intestinal tissues were cut into smaller pieces (~0.5 cm) and digested within RPMI-1640 medium (Gibco, Waltham, MA, USA) containing DNase I (Solarbio, Beijing, China) (150 µg/mL) and collagenase VIII (Gibco, Waltham, MA, USA) (200 U/mL) on a shaker at 37 °C for 70 min. The digested tissues were subject to vigorous shaking by hands and removal of large debris through a 70 µm cell strainer. The obtained cell suspension was centrifuged in a Percoll gradient at 800 × g for 20 min and the mononuclear T lymphocytes were collected at the 40%/80% interphase.

For the flow cytometry analysis, the cell phenotyping was analyzed using anti-CD16/32, anti-CD3, anti-CD4, anti-CD25, anti-CD103, anti-FoxP3, and anti-Helios. The above colonic lymphocytes (2 × 10^6^ cells) were first stained with Fixable Viability Dye (eBioscience; Catalog No.: 65-0866-14; at a final concentration of 1 µL/mL) for 30 min. Anti-CD16/32 (Elabscience, clone 2.4G2; E-AB-F0997A; 10 µg/mL) antibody was used to block the non-specific binding to Fc receptors before all surface staining. For the cell surface staining, lymphocyte cells were incubated with antibodies of anti-CD3-PerCP-Cyanine5.5 (Elabscience, clone 17A2; E-AB-F1013J; 50 µL/mL), anti-CD4-ER780 (Elabscience, clone GK1.5; E-AB-F1097S; 50 µL/mL), anti-CD25-APC (Elabscience, clone PC-61.5.3; E-AB-F1102E; 50 µL/mL) and anti-CD103-PC7 (Elabscience, clone M290; E-AB-F1090H; 10 µg/mL) for 1 h according to the manufacturer’s recommendations. Subsequently, the cells were fixed and permeabilized with a Foxp3 staining buffer kit (eBiosciences; 00-5523) and stained with anti-Foxp3-PE (Elabscience, clone 3G3; E-AB-F1238D; 50 µL/mL) and anti-Helios-FITC (eBioscience, clone 22F6; 11-9883-82; 2.5 μg/mL) for 1 h. The results were acquired on a flow cytometer (Beckman Coulter CytoFLEX-LX) and shown using FlowJo software. The gating strategy for flow cytometry is shown in Supplementary Fig. 7.

### qPCR measurement of *foxp3* gene expression

Total RNA of proximal colonic tissue was extracted using TRIzol kit (Invitrogen, Canada) according to the manufacturer’s manual. The cDNA was synthesized with OligoDT primers using GoScript Reverse Transcription System (Promega, USA) kit (Tm = 25 °C). The expression levels of the gene *foxp3* in colonic tissues were measured using qPCR with primers described in Supplementary Table 1. The qPCR procedure was performed using the SYBR Green-based method of GoTaq® qPCR Master Mix Kit (Promega, USA) according to the manufacturer’s recommendation (Tm = 60 °C) on a ‘Applied Biosystems 7500’ Real-Time PCR system (ABI, Thermo Fisher Sci). The relative expressions were calculated using the 2 ^-ΔΔ^ CT method and the housekeeping gene *β‑actin* was used for internal control to normalize the expression of the target gene.

### Isolation, culture, and administration of *Lactobacillus reuteri*

Mice feces were collected from the mice at day 90 post-*Emu* infection, homogenized in sterile PBS, serially diluted, and plated onto MRS or LB agar. The plates were incubated in an anaerobic chamber at 37 °C for 24 h. The bacterial colonies that developed on the plates were picked and streaked on fresh MRS agar plates by dilution streaking to obtain single colonies. The typical colonies of *Lactobacillus* spp. and *Escherichia* spp. were picked and anaerobically cultured on MRS or LB broth at 37 °C for 24 h. The isolates were identified by using 16 S rRNA gene sequencing. Briefly, the genomic DNA of each clone was extracted using a QIAamp PowerFecal Pro DNA Kit (QIAGEN, Germany), according to the manufacturer’s instructions and further sequenced for the 16 S rRNA gene using general primers (TSINGKE Biological Technology Company, China). The produced sequences were searched using BLASTn against NCBI ‘nr’ database to make a taxa assignment. Only one *L. reuteri* strain and one *E. coli* strain were identified. The isolate was stored at −80 °C in 30% glycerol with the medium until use.

The identified *L. reuteri* and *E. coli* isolates were recovered in MRS medium or LB medium. *B. xylanisolvens* (CCUG53782T) was purchased from MingZhoubio Co., LTD (Ningbo, China) and *B. intestinalis* (BNCC353580) was purchased from Jiachu Biological Engineering Co., LTD (Shanghai, China). These two bacteria were inoculated with Gifu Anaerobic Medium in an anaerobic chamber at 37°C. The culture of the bacteria was harvested at the exponential phase (with an initial OD600 of 0.04) and freshly prepared before administration to mice. Each mouse was pretreated with the above antibiotic cocktails for 2 weeks and then orally gavaged with 2 × 10^9^ CFU of each bacterium isolate in 200 μL PBS or PBS alone (control) daily for 2 weeks. The mice were euthanized at 4 weeks post the *L. reuteri, B. xylanisolvens, B. intestinalis and E. coli* treatment and subject to a flow cytometry analysis.

To compare colonization of the *L. reuteri* isolate in Balb/c and nude mice, the mice were pre-treated with the antibiotic cocktail once at day 60 post-*Emu*-infection and gavaged for three consecutive days from day 62 to 64. The total genomic DNA of samples was extracted and DNA concentration was quantified as described above. To quantify the level of *L. reuteri* in the feces of the mice, qPCR (Primers described in Supplementary Table 1; targeting 16 S rRNA gene^45^) was used with the following working system: DNA 100 ng (50 ng/μL), 10 uL SYBR Green mix, 0.4 μL each primer (10 μmoL) and RNA-free water 7.2 μL in a total volume of 20 μL. The qPCR procedure was conducted with an annealing temperature at 60 °C using GoTaq® qPCR Master Mix Kit (Promega, USA) using an ABI7500 machine (ABI, USA). The analysis ran alongside a serially-diluted pool of all samples to be analyzed to create a standard curve of Ct value versus 16 S rRNA gene level in arbitrary units^7^. Data for each sample (set up in triplicate) are presented as the mean expression level in arbitrary units. The levels of other microbes in feces were measured by the same qPCR procedure mentioned above, using primers described in Supplementary Table 1.

### Shotgun metagenomic sequencing and analysis

Genomic DNA was extracted from stool using the fecal DNA extraction kit (magnetic soil and stool DNA kit, Tiangen) following the manufacturer’s recommendations. The DNA libraries of the samples were constructed using NEBNext® Ultra™ DNA Library Prep Kit for Illumina (NEB, USA) following the manufacturer’s recommendations. The library quality was assessed on the Qubit 2.0 fluorometer (Thermo Scientific) and Bioanalyzer 2100 system (Agilent), and then sequenced on a NovaSeq6000 platform (Illumina). The produced raw reads (paired-end; 150 bp) were removed with barcodes and low-quality reads (reads with N bases of more than 10% and reads with low-quality bases [Q ≤ 5] of more than 50%) using fastp^46^. The potential contamination from the host (mouse, the genome version: GRCm38.p4) was detected and removed using KneadData (v0.10.0)^47^ (settings: -t 30) and the ‘trimmomatic’ function in KneadData was used for the second filtering of the reads with more strict parameters (SLIDINGWINDOW:5:20 LEADING:3 TRAILING:3 MINLEN:50). Taxonomic annotation of each sample was generated using Metaphlan3 (v3.1.0)^47^ based on the reference database of ‘mpa_v31_CHOCOPhlAn_201901’. Functional and pathway composition was calculated with HUMAnN2^48^ using the UniRef90 database and UniPathway database with default settings (‘full_chocophlan_plus_viral.v0.1.1’ and ‘uniref90_annotated_1_1’). Differential analysis of each taxon at the species level was performed by the analysis of compositions of microbiomes (ANCOM) method with default settings in the ANCOMBC package (v1.64)^49^. The abundance of each pathway in each species was normalized with Counts Per Million (CPM).

### Quantification of short-chain fatty acids (SCFAs)

Fecal samples were sent to Shanghai Lu-Ming Biotech Co., Ltd. (Shanghai, China) for GC-MS analysis of SCFAs. Briefly, 20 mg freeze-dried samples were diluted with 400 μL ice-cold ethanol, incubated at −20 °C for 2 min, and ground with a grinder (60 Hz, 2 min). The samples were subject to ultrasonic treatment for 20 min in an ice-water bath. The impurities in supernatants were removed by centrifuging at 13,523 × g at 4 °C for 10 min. The obtained supernatants were then filtered through a 0.22 μm organic phase pinhole filter and transferred into a new 2 ml glass vial for GC (GAS chromatography) analysis on an Agilent 7890B-5977B gas chromatograph system (Agilent Technologies, CA, USA), in which a DB-WAX capillary column (30 m × 0.25 mm × 0.25 μm) (Agilent, Folsom, CA, USA) was used. The SCFAs in each sample were identified and quantified by a flame ionization detector. The data were analyzed using the Masshunter software (Agilent, USA). The concentration of each SCFA was evaluated with the linear regression equations from the corresponding standard curves.

### Statistical analysis

Statistical analyses were performed using R (v4.2.0) and Prism 9 software (GraphPad Software, La Jolla, CA). The abundances of taxa, alpha diversity and pathways were compared between groups using the Wilcoxon rank-sum test (two-sided; confidence level of 0.95). PCA analyses of β-Diversity and pathways were measured using Permutational multivariate analysis of variance (PERANOVA) with the ‘Adonis2’ function in ‘vegan’ package (v2.6-4) of R. A two-tailed unpaired Student’s *t*-test determined other statistical significances for independent samples. The *p*-values for the multiple hypothesis tests were corrected by the False Discovery Rate (*FDR*) (‘Benjamini-Hochberg’ method). Values of *P* < 0.05 or *FDR* < 0.05 were considered statistically significant.

### Reporting summary

Further information on research design is available in the Nature Research Reporting Summary linked to this article.

## Supplementary information

Supplementary figures and tables

Reporting Summary

Supplementary Data 1

## Data Availability

The metagenomic data in this study have been deposited at NCBI under the BioProject: PRJNA892774. Other data that support the findings of this study are available from the corresponding authors upon reasonable request.

## Code availability

Microbial and gene abundance tables and the code for analyses in this study are available on GitHub (https://github.com/Axolotl233/Emu-gut_microbiome).
