# Supplementary material for: Interaction between tissue-dwelling helminth and the gut microbiota drives mucosal immunoregulation
Source: NPJ Biofilms Microbiomes. 2023 Jun 24;9:43. doi: 10.1038/s41522-023-00410-7 (PMC10290639; doi:10.1038/s41522-023-00410-7)
Supplement: Supplementary file 1 — Supplementary figures and tables [file 41522_2023_410_MOESM1_ESM.pdf]

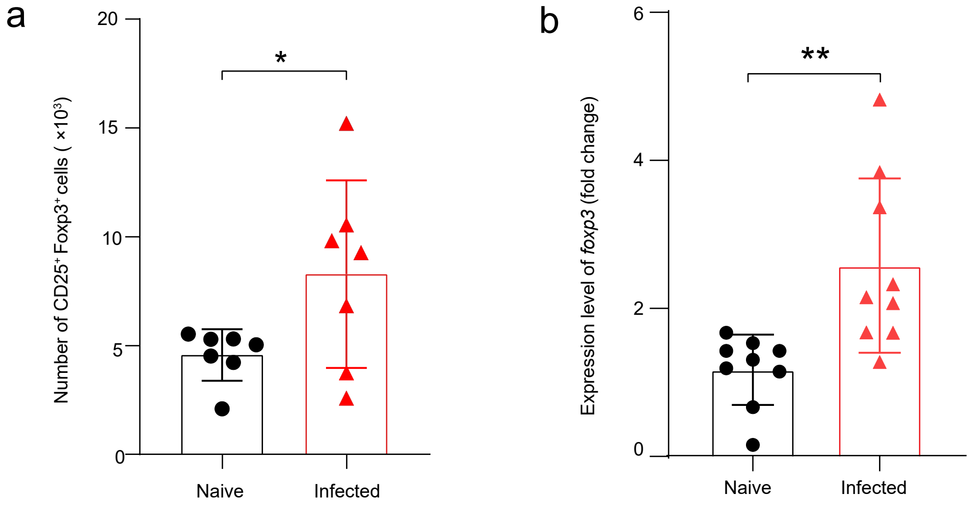

Supplementary Figure 1. The cell number of CD25⁺Foxp3⁺ Tregs is increased after *Emu* infection. (a) On day 90 post-infection of *Emu*, the naive (Naive) and infected (Infected) mice were euthanized and subject to the flow cytometry analysis of colonic $CD25^+Foxp3^+$ T cells in lamina propria. (b) The expression level of *foxp3* in proximal colonic tissue was measured using qPCR. Flow cytometry data are representative of at least 5 mice per group. Experments were repeated 2 times with similar results. The data shown are from a representative experiment (mean ± SD shown). *$P < 0.05$, **$P < 0.01$ using the two-sided *Student t-test* (n = 7 mice per group for panel a; n = 9 mice per group for panel b).

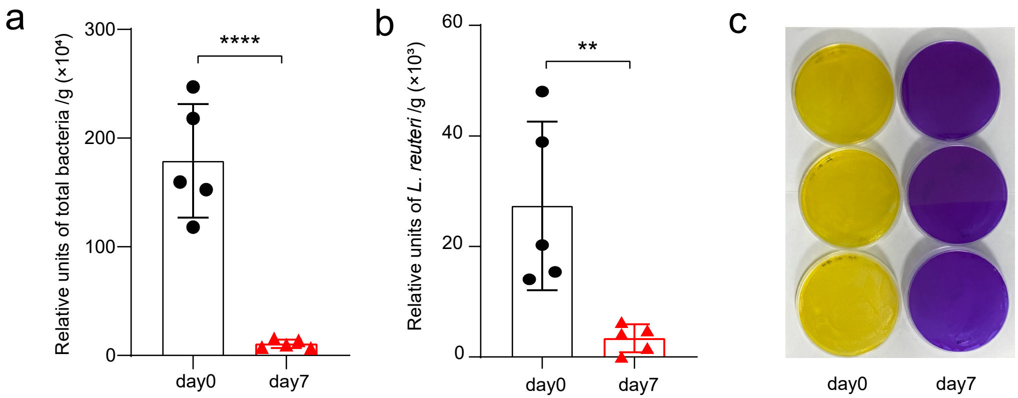

Supplementary Figure 2. The bacteria load in the feces of the mice treated with ABX. (a) The relative units for total bacteria. (b) The relative units for *L. reuteri.* (c) The test for the ability to produce SCFAs on pH-sensitive Bromocresol purple agar (BCP) plates. $^{**}P < 0.01$, $^{****}P < 0.0001$ using the two-sided *Student t-test* (n = 5 mice per group).

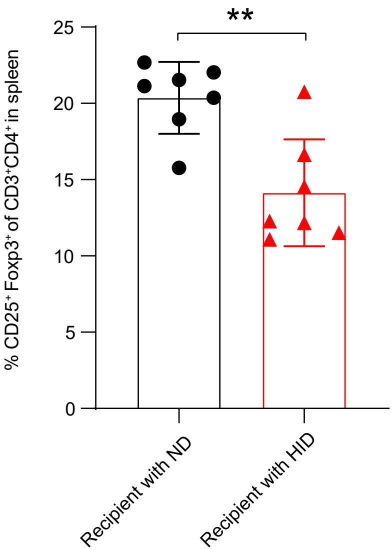

Supplementary Figure 3. The proportion of splenic $CD25^+Foxp3^+$ Treg cells. The Treg cells in $CD3^+CD4^+$ immune cells were analyzed using flow cytometry for the recipient mice after the FMT experiment. Experiments were repeated 2 times with similar results. The data shown are from a representative experiment (mean ± SD). $^{**}P < 0.01$ using the two-sided *Student t-test* (n = 7 mice per group).

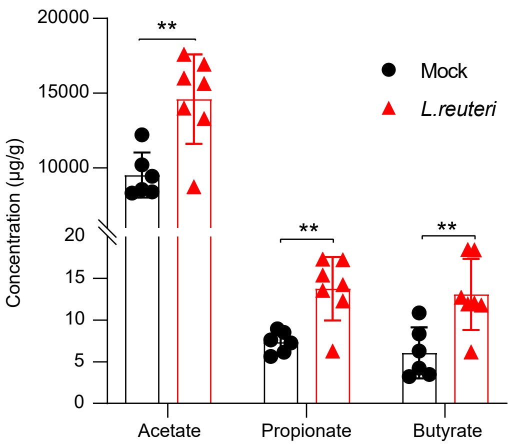

Supplementary Figure 4. The concentrations of SCFAs in feces for the mice gavaged with the *L. reuteri* isolate. **$P < 0.01$ using the two-sided *Student t-test* (n = 6-7 mice per group).

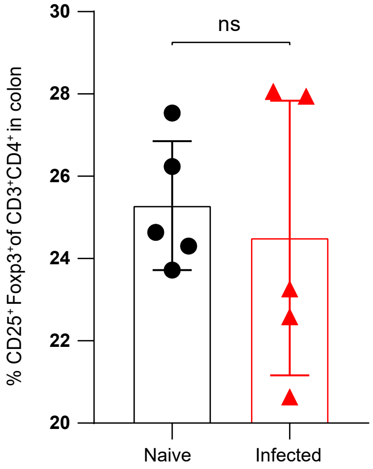

Supplementary Figure 5. The proportion of Tregs in mice at 6 weeks post-*Emu* infection. At week 6 post-infection of *Emu*, the mice were euthanized and subject to flow cytometry analysis of colonic (lamina propria). The proportions of colonic $CD25^{+}Foxp3^{+}$ T cells in the $CD3^{+}CD4^{+}$ cell population are shown for the representative of each group. ns, not significant (n = 5 mice per group).

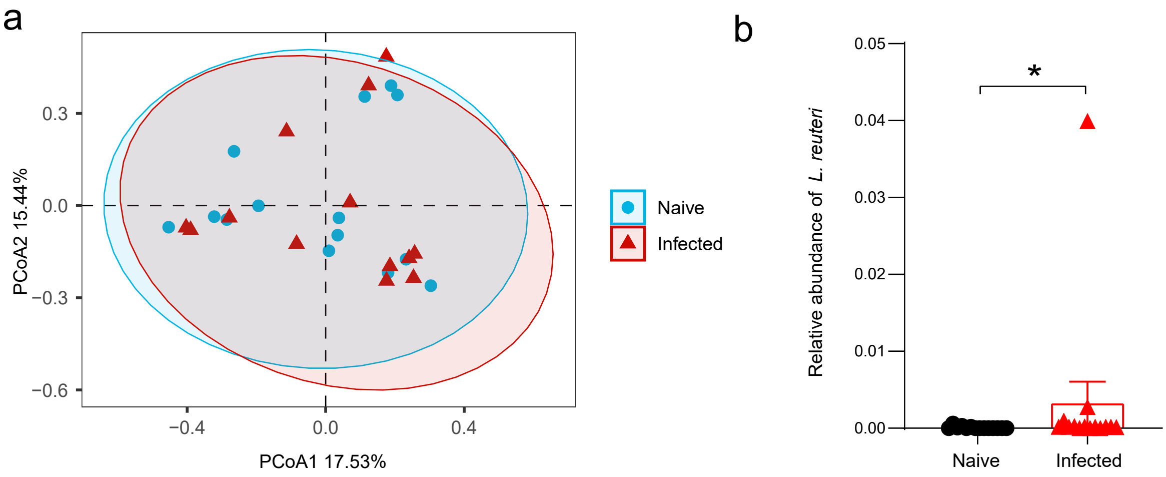

Supplementary Figure 6. The 16S rRNA metagenomic analysis of fecal samples. Stool samples were collected from the naive and *Emu*-infected mice at week 6 post-infection and analyzed using 16S rRNA sequencing (n = 13 mice for naive group; n = 12 mice for infected group). (a) Principal coordinates analysis (PCoA) for the gut microbiome of the samples was performed based on the relative abundance of each taxon at the species level ($P > 0.05$). (b) The relative abundance of *L. reuteri* in 16S rRNA metagenomic analysis. The significance of PCoA analysis for panel a was determined by Permutational multivariate analysis of variance (PERANOVA). *$P <$ 0.05 using the two-sided *Student t-test* for panel b.

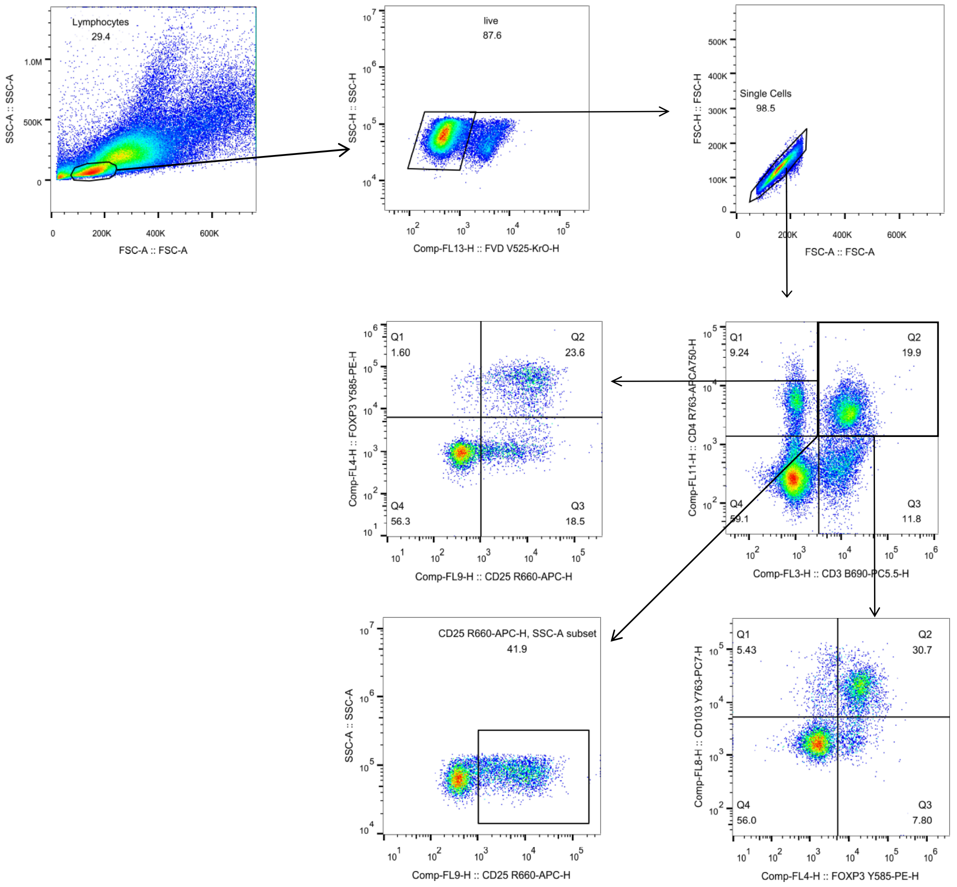

Supplementary Figure 7. The gating strategy for flow cytometry. Percentages of CD25$^+$Foxp3$^+$ or Foxp3$^+$ Treg cells among CD3$^+$CD4$^+$ T cells and CD103$^+$ Foxp3$^+$ Treg cells among CD3$^+$CD4$^+$ T cells analyzed in this article. Forward and side scatter gating was used to remove debris and select lymphocytes. Alive cells were then selected by negativity for viability dye. Forward scatter height vs. area to enrich for single cells. The following gating steps used positive or negative staining controls to identify the specific cells of interest. CD4$^+$ T helper cells were then selected by CD3$^+$ vs. CD4$^+$ dot plot. Treg cells were identified by CD25$^+$ vs. Foxp3$^+$ or CD103$^+$ vs. Foxp3$^+$ dot plot, gated from CD4$^+$ T helper cells.

Supplementary Table 1. Primer sequences used in this study.

Target species/gene	Sequences (5'-3')
Total bacteria (16S rRNA)	F: CGGYCCAGACTCCTACGGG
	R: TTACCGCGGCTGCTGGCAC
L. reuteri	F: CAGACAATCTTTGATTGTTTAG
	R: GCTTGTTGGTTTGGGCTCTTC
B. intestinalis	F: AGCATGACCTAGCAATAGGTTG
	R: ACGCATCCCCATCGATTAT
B. xylanisolvens	F: ACGCTCGGATCCTCCGTATT
	R: AGGATGACTGCCCTATGGGT
E. coli	F: CATGCCGCGTGTATGAAGAA
	R: CGGGTAACGTCAATGAGCAAA
foxp3	F: AGCAGGAGAAAGCGGATACC
	R: TCTGTGAGGACTACCGAGCC
β-actin	F: ACGTTGACATCCGTAAAGAC
	R: GAAGGTGGACAGTGAGGC
